# Thoughts on the Abuse of Dental Practice

**Published:** 1840-07

**Authors:** John C. McCabe


### THOUGHTS ON THE ABUSE OF DENTAL PRACTICE.

[ BY JOHN C. McCABE. ]

It is to be lamented, that in this age of scientific discovery, and philosophic research, in proportion to the improvements of the day, is the prevalence of imposture.

It is only necessary for valuable discoveries to be made, or new and popular theories to be broached, in order to the almost magical raising up of a host of miserable pretenders, whose chief merit is the *impudence* with which they assume the honors which properly and legitimately belong to others.

Could the celebrated Gall, or the renowned Spurzheim have imagined that the interesting science which they labored to establish, was destined to be peddled about like wooden nutmegs, or horn gun flints; and *character* retailed out at a dollar per head, they would possibly have confined their labours to some other mine of thought, were one more attractive, but less solid, might have pleased without exciting the cupidity of the charlatan.

No science has, perhaps, suffered as much from gross outrageous charlatanism, as dental surgery; at a period too, when its principles, as a science of a high order of surgery is acknowledged by the most intelligent of the medical faculty.

Indeed, it may, in some degree, be owing to the respectability which some of its distinguished professors have succeeded in establishing for it, that so many pretenders have gone forth into the dental field. Not satisfied with "*rooting* up the tares, but the wheat also;" or in other words, not content with removing carious and diseased teeth, but pulling *all* that come in the reach of their mallets, forceps and keys.

He who yesterday wielded the blacksmith's hammer with unaspiring content, to day puts forth a sign, bearing on it in great flaring letters, "SURGEON DENTIST;" and he who imagined at one time a tailor's shop board little less than the throne of the Cæsars, now turns up his *professional* nose at his "ninth proportion" of an old associate, and "thanks him, if he has occasion to address him, to please remember to call him Doctor."

This may to some wear the appearance of exaggeration, but the writer is acquainted with *facts* connected with the assumption of the practice by grossly ignorant men, which, if the result was not calculated to jeopardize life itself, would excite the risible faculties of the most imperturbably grave.

He remembers, some few years since, having been shown a letter from an ignorant fellow, who had formerly been a bar-keeper at a little inn, to a gentleman in this city: the individual had left here with a drove of horses, like Milton's Adam :—

> " The world before him, where to choose a place of rest,
> And Providence his guide."

Six months after he had left Richmond, the letter alluded to was written, —and as nearly as recollected, ran as follows, "*verbatim, &c.*"—

" Deer Sur

I take my pen in hand to Inform you of how I am duing. I have turned dentist and am making munney I have a mity nice office in this place, and keeps a Sulkey I am very fond of drawing teeth and meen to lurn how to make seehorse teeth. this is a better prefeshun than mayking Cocktales at hapence a glass. Yew must rite to meen at this place.

I reemayn your humble survant," &c.

Now this poor fellow gloried, he says, in *drawing teeth*, and I must confess, if he *drew* them with the same self-possession and confidence in his own abilities, with which he *drew* the *picture* of his success, he must have succeeded to a hair.

This, however, is one among many similar cases,—no doubt, and the reason perhaps, why some enter without sufficient preparation into the profession, is the prevailing opinion, that *mechanical* ingenuity is the *only* requisite to the making of a dentist.

No opinion can be more erroneous.—As well might it be said that the surgeon who trephines, sets joints, splices limbs, and performs the various duties of his appropriate branch of the profession, could learn his business in a carpenter's shop or a joiner's—and yet some physicians, as ignorant of the science of dental surgery as those spoken of, have been heard to say, and the impression is a general one, except with individuals who know to the contrary, that drawing a tooth is the *easiest* thing in the world—" *any body* can draw a tooth."

Impressions thus conveyed, persons take it for granted, that these are facts, and submit their mouths to the manipulations of the ignorant pretender with the same confidence in his ability which they would manifest towards the most scientific; and the teeth—those delicate organs—are operated upon by men who are as ignorant of their physiology and the anatomy of the parts, as the blindest heathen is of the existence of the great word of truth.

This is a crying evil, acting with two-fold prejudice—affecting the profession, by the disgrace which this wretched bungling sometimes attaches to it, and periling the lives of those who are simple enough to be imposed upon by their pretensions : and it is high time that the public and the profession should unite in devising means for the suppression of such impostures.

The Faculty of Baltimore have put the ball of reform in motion, and if sustained, will "reform it altogether;" and it must afford the intelligent dentist infinite satisfaction to know that his profession, though disgraced by some of the worst specimens of humanity, numbers some of the most intellectual and scientific in the country. Let their efforts in the cause be sustained, *their publication supported*, and the reform so much needed will be brought about. These "poor practitioners" will have to turn their attention to some other *science*—hoeing corn, or planting potatoes and removing *stumps* of *trees*, while the *legitimate* professors of the art will receive that encouragement and support which their labors deserve.

It is necessary that there should be some *test* of qualification by bodies qualified and regularly appointed for the duty; and if every state was to enact a law of this character, the profession would be protected against the assumptions of those pretenders in the cause, and its ranks thinned of many who in time past have availed themselves of the name and its benefits, and yet were ignorant of the first principles of the profession.

Lest it might be supposed that the writer designed *particularizing*, he begs leave, most positively to object to such a conclusion. All the dentists of his acquaintance, he is proud to say, are ornaments to the profession; and the individual, whose classic letter is here introduced, has long since given up the making of *see horse* teeth and gone to *raising hogs*. That the evils here spoken of *do* exist, the profession will bear me out in asserting, and that they should be arrested, public opinion will not gainsay.

It may argue presumption in one so young, and so inexperienced in preparing "paper bullets o' the brain" to thus attack an error which older and worthier champions in the ranks, have passed by as unworthy of notice; but as it is sometimes necessary for an employer to leave to his apprentice the task of removing the *rubbish*, so he wishes rather to be considered as acting, than as playing the censor where others have feared, or failed to rebuke the evil.

